# Medullary Respiratory Circuit Is Reorganized by a Seasonally-Induced Program in Preparation for Hibernation

**DOI:** 10.3389/fnins.2019.00376

**Published:** 2019-04-26

**Authors:** Thomas L. Russell, Jichang Zhang, Michal Okoniewski, Felix Franke, Sandrine Bichet, Andreas Hierlemann

**Affiliations:** ^1^Department of Biosystems Science and Engineering, ETH Zürich, Basel, Switzerland; ^2^Scientific IT Services, ETH Zurich, Zurich, Switzerland; ^3^Friedrich Miescher Institute for Biomedical Research, Department of Histology, Basel, Switzerland

**Keywords:** microelectrode array, electrophysiology, hibernation, respiratory rhythms, ventral respiratory group, medulla, Syrian hamster, pre-Bötzinger complex

## Abstract

Deep hibernators go through several cycles of profound drops in body temperature during the winter season, with core temperatures sometimes reaching near freezing. Yet unlike non-hibernating mammals, they can sustain breathing rhythms. The physiological processes that make this possible are still not understood. In this study, we focused on the medullary Ventral Respiratory Column of a facultative hibernator, the Syrian hamster. Using shortened day-lengths, we induced a “winter-adapted” physiological state, which is a prerequisite for hibernation. When recording electrophysiological signals from acute slices in the winter-adapted pre-Bötzinger complex (preBötC), spike trains showed higher spike rates, amplitudes, complexity, as well as higher temperature sensitivity, suggesting an increase in connectivity and/or synaptic strength during the winter season. We further examined action potential waveforms and found that the depolarization integral, as measured by the area under the curve, is selectively enhanced in winter-adapted animals. This suggests that a shift in the ion handling kinetics is also being induced by the winter-adaptation program. RNA sequencing of respiratory pre-motor neurons, followed by gene set enrichment analysis, revealed differential regulation and splicing in structural, synaptic, and ion handling genes. Splice junction analysis suggested that differential exon usage is occurring in a select subset of ion handling subunits (ATP1A3, KCNC3, SCN1B), and synaptic structure genes (SNCB, SNCG, RAB3A). Our findings show that the hamster respiratory center undergoes a seasonally-cued alteration in electrophysiological properties, likely protecting against respiratory failure at low temperatures.

## Introduction

Environments with large seasonal variances in temperatures often create regular conditions of resource scarcity. Many animals adapted to living in such regions have evolved the ability to periodically engage bouts of torpor, whereby they lower their body temperatures, subsequently lowering metabolism and conserving energy. In deep hibernating rodents, these phases of profound cooling can last days to weeks, whereupon the animal’s body temperature will fall to near freezing and then recover to full euthermic temperature. The torpor cycle is particularly interesting because it exposes the animal to the possibility of respiratory arrest, yet the animal manages to remain in a state of respiratory fidelity.

Even though hibernators possess active metabolic controls for avoiding hypoxia-ischemia during body temperature drops [as well as other mechanisms for lessening its effects such as blood shunting (Frerichs et al., 1994) and elevated hemoglobin O_2_ affinity (Maginniss and Milsom, 1994)], breathing rhythms must still be maintained, albeit at a slower rate. In adult rats, medullary temperatures below 16.6°C will induce respiratory arrest (Arokina et al., 2011). For homothermic mammals, the lack of temperature robustness in the breathing circuit itself poses the risk of runaway hypoxia and death. Hibernators likely posses unique neuronal circuitry characteristics that allow breathing rhythm generation at low temperatures and protect against respiratory arrest.

The presence of such a mechanism would not be unprecedented, although investigations of seasonal changes in mammalian brain electrophysiology are surprisingly scarce. Jinka et al. (2011) Illustrated an adenosine receptor-mediated seasonal “priming” of the central nervous system in ground squirrel. Similarly, Barrett et al. (2009) showed that exposing Siberian hamsters to a prolonged short photoperiod increases neuronal activity in the arcuate nucleus of the hypothalamus, mediated by changes in expression of the histamine H3 receptor (Herwig et al., 2012). The hypothalamus incidentally is another brain region which remains active during hibernation likely due to its role in timing of bouts (Heller and Ruby, 2004; Bratincsák et al., 2009).

**FIGURE 1:**
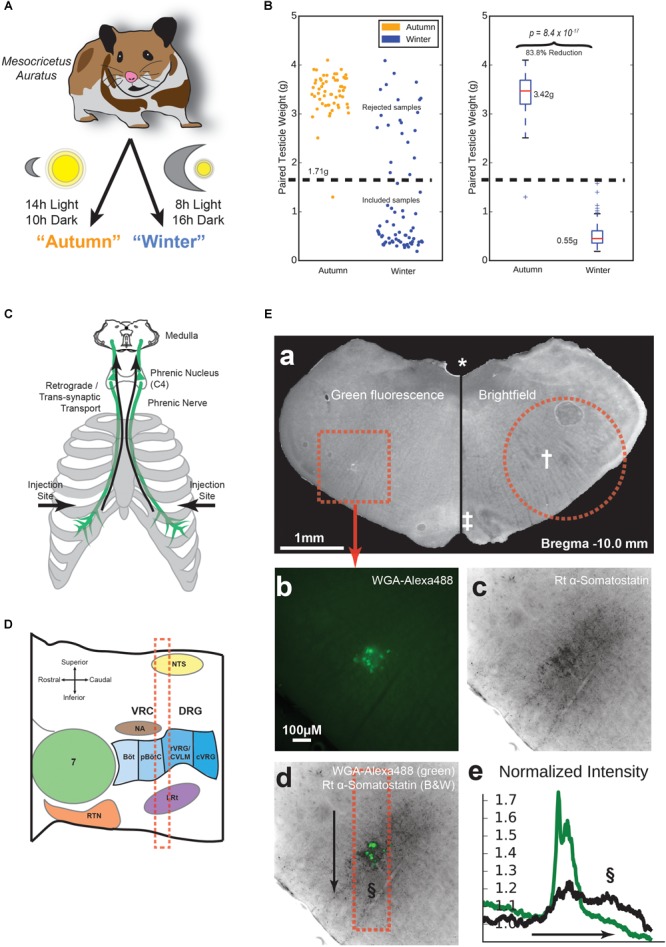
Animal preparation and location of the pre-Bötzinger complex (preBötC). **(A)** Experimental groups. Hamsters were either kept at 14h/10h light/dark from age 3 to 11 weeks, or 8h/16h light/dark during the same age range, to make non-adapted “Autumn” and adapted “Winter” groups, respectively. **(B)** Testicular recession in winter hamsters. After euthanasia, testicles were dissected and weighed. Winter hamsters not showing at least a 50% reduction in paired testicle weight were excluded from further experimentation. **(C)** Tracing method. Approximately 100 μg in 10 μl of Wheat Germ Agglutinin (WGA) in PBS was injected into the pleural cavity under the fifth intercostal space. WGA was taken up by the phrenic nerve and transported retrogradely to the phrenic nucleus in spinal cord C4 and further transported trans-synaptically into the medulla. **(D)** Parasagittal schematic of the medullary area under study. Landmarks according to Feldman, adapted from (Stornetta, 2009). The red dashed box indicates approximate acute slice location for subsequent experiments. **(E)** Labeling and immunohistochemical staining. **(a)** Representative medullary brain slice (100 μm thickness). Left: fluorescence image containing WGA-Alexa488 labeling of respiratory pre-motor neurons. The dotted square indicates the area shown in **(b–d)**. Right: Brightfield image of the contralateral side of the same slice. The dotted circle indicates approximate boundary of the tissue punch taken for electrophysiological recording. Landmarks: (^∗^) fourth ventricle, (†) Nucleus Ambiguus (NA), (‡) Inferior Olive. **(b)** Raw intensity map of green fluorescence, indicating WGA-Alexa488 labeling. **(c)** Raw intensity map of fluorescence indicating labeling by rat anti-Somatostatin antibody. **(d)** Overlay of images from **(b)** and **(c)**. The dotted box indicates the area of measured fluorescence intensity values in **(e)**, along the axis indicated by the arrow, sweeping from top to bottom. **(e)** Normalized fluorescence intensity values along the arrow axis in **(d)** for WGA-Alexa488 (green) and Somatostatin labeling (black). Values are normalized to a parallel area near the right of the image. The §symbol indicates the putative location of the preBötC in **(d)** and **(e)**.

In this study, we explore the possibility that seasonal cues enact a genetic program to protect a deep hibernator against the threats posed by respiratory arrest. We used the Syrian hamster (*Mesocricetus auratus*), which can be cued by short day length to enter a physiological state that is a prerequisite for hibernation to occur (Gaston and Menaker, 1967; Frehn and Liu, 1970; Ueda and Ibuka, 1995). By comparing short day-length adapted “winter” hamsters with non-adapted “autumn” hamsters (Figure 1A), we were able to measure how this preparatory signal affected circuit functionality and cell viability in an area of the brain with critical functionality: the respiratory center.

We reasoned that since the respiratory center is absolutely critical for life, it must sustain breathing at low temperatures to prevent runaway hypoxia. This makes it plausible that our experiments would uncover restructuring of respiratory circuits and synapses, representing an altogether novel means by which the brain could protect itself during the hibernation process. Since breathing rhythms are generated in the pre-Bötzinger complex (preBötC) (Smith et al., 1991; Feldman and Del Negro, 2006; Feldman, 2014), and can be seen to sustain *in vitro* in mammalian models (Smith and Feldman, 1987; Smith et al., 1990; Funk and Greer, 2013), we can easily assess the performance of the respiratory circuit at a wide range of temperatures for both winter-adapted and non-adapted animals. Additionally, the availability of RNA sequencing allows us to look into the gene expression changes that occur in this area during the change of seasons.

## Materials and Methods

### Animals

All procedures were approved by the Basel-City Cantonal Veterinary Authority. Male Syrian hamsters (*Mesocricetus auratus*) were received from the supplier (Janvier Labs, Le Genest-Saint-Isle, France), who housed them under long day conditions (LD, 14 h:10 h). After receiving the animals, they were housed in the institutional animal facility under short day conditions (SD, 8 h:16 h) for 1 week prior to beginning the experiments (“autumn” control condition), or for 8 weeks to induce physiological adaptations for hibernation (“winter” condition), Figure 1A. Age of the animals ordered was controlled such that all animals were 11 weeks old at the time of sacrifice. After killing, testicles were dissected and weighed. Of all hamsters housed under short-day conditions, 74.6% showed a testicle weight reduction of at least 50% below the average testicle weight of the control group (autumn) and were included in the hibernation-adapted (winter) group (Figure 1B).

### Labeling of Respiratory Neurons

In order to label the respiratory region in the medulla for immunohistochemistry or RNA sequencing, some animals received an injection of a fluorescent, trans-synapic, retrograde tracer. These animals were anesthetized with isoflurane and bilaterally injected 10 μl of PBS containing 10 mg/ml Alexa488 conjugated Wheat Germ Agglutinin (WGA) (Thermo-Fisher, Reinach, Switzerland) into the intrapleural cavity beneath the fifth intercostal space of the rib cage (Figure 1C) as indicated in Buttry and Goshgarian (2014, 2015). Over the course of 48 h, the WGA was taken up by phrenic nerve efferents and retrogradely transported to the phrenic nucleus in the spinal cord. Further trans-synaptic retrograde transport took place and ultimately labeled cell bodies in the medulla, which presumably control breathing rhythms. After 48 h, tissue was collected in the same manner as animals used for electrophysiology, as follows.

### Tissue Preparation

Animals were anesthetized with 30 mg/kg tiletamine-zolazepam/10 mg/kg xylazine and killed by decapitation. The brainstem was quickly removed after sacrifice and sliced in 300-μm-thick sections on a Compresstome (Precisionary Instruments, Greenville, NC, United States) in 4°C cutting solution (93 mM NMDG, 2.5 mM KCl, 20 mM Hepes, 30 mM NaHCO_3_, 1.2 NaH_2_PO_4_, 5 mM Na-Ascorbate, 2 mM Thiourea, 3 mM Na-Pyruvate, 0.5 mM CaCl_2_, 10 mM MgSO_4_, 1–10 mM Glucose, pH 7.3). The strongest labeling was located 200–300 μm caudal to the NA (Figure 1D). We used a 1.8-mm-diameter tissue punch from the 300-μm-thick slice caudal to the Nucleus Ambiguus (NA). Slices were placed into 33°C oxycarbon (95% O_2_, 5% CO_2_)-bubbled recovery solution (115 mM NaCl, 3 mM KCl, 10 mM Hepes, 30 mM NaHCO_3_, 0.5 NaH_2_PO_4_, 2 mM Na-Ascorbate, 2 mM Thiourea, 2 mM Na-Pyruvate, 2 mM CaCl_2_, 1 mM MgSO_4_, 1–10 mM Glucose, pH 7.3) for 1 h before recording. Testicles were dissected and weighed to confirm induction of winter adaptation.

### Immunohistochemistry

For animals used to confirm the location of the preBötC with respect to labeling, animals were deeply anesthetized with 30 mg/kg tiletamine-zolazepam/10 mg/kg xylazine and transcardially perfused with ice-cold PBS followed by ice-cold 1% formalin PBS. Brains were then extracted and placed into 4% formalin PBS overnight at 4°C. Brains were placed into PBS for 2 days, then sliced in 100 μm thick slices using a Compresstome. Free-floating slices were then permeablized with 0.25% Triton X-100 PBS for 10 min, followed by a 30 min block with 1% bovine serum albumin in 0.1% Tween-20 in PBS (BSA-PBST). Slices were then incubated for 4 days at 4°C in BSA-PBST containing the antibody for somatostatin (1:100, ab30788, Abcam, Cambridge, United Kingdom). After washing with PBST, slices were incubated with a secondary antibody (anti-Rat, 1:400, Jackson ImmunoResearch Europe, Suffolk, United Kingdom) at room temperature for 1 h. Slices were washed and mounted with Permount (Fisher Scientific, Reinach, Switzerland), and imaged on an inverted fluorescence microscope (Nikon).

### Viability Assays

Acute brain slices were prepared as indicated above, but with 1.2-mm diameter punches instead of 1.8 mm. After 30 min of bubbling in recovery solution, punches were placed into a custom-made chamber that allowed punches to be individually incubated in 1 ml of recovery solution with oxycarbon bubbling. Punches were loaded with 10 μM Calcium Green-AM (Life Technologies) and 5 μM CellRox far-red oxidative stress label (Life Technologies). After another 20 min, a live-dead label was added to the chambers and incubated for an additional 10 min. Punches were then placed into fresh recovery solution in a 6-well plate, sealed, and imaged on an inverted microscope (Nikon). Focus was adjusted to bring the maximum number of red channel (Propidium iodide) cells into focus. Each color was imaged for 0.5 s. Punches, plus 100 μl recovery solution, were combined with 100 μl 3D adenosine triphosphate (ATP) luminescence assay (Promega) in separate wells of a 96-well plate. Punches were dissociated by pipetting every 10 min for 30 min. One hundred μl of each sample was pipetted into a separate well and luminescence was measured (Tecan), along with an ATP standard curve.

**FIGURE 2:**
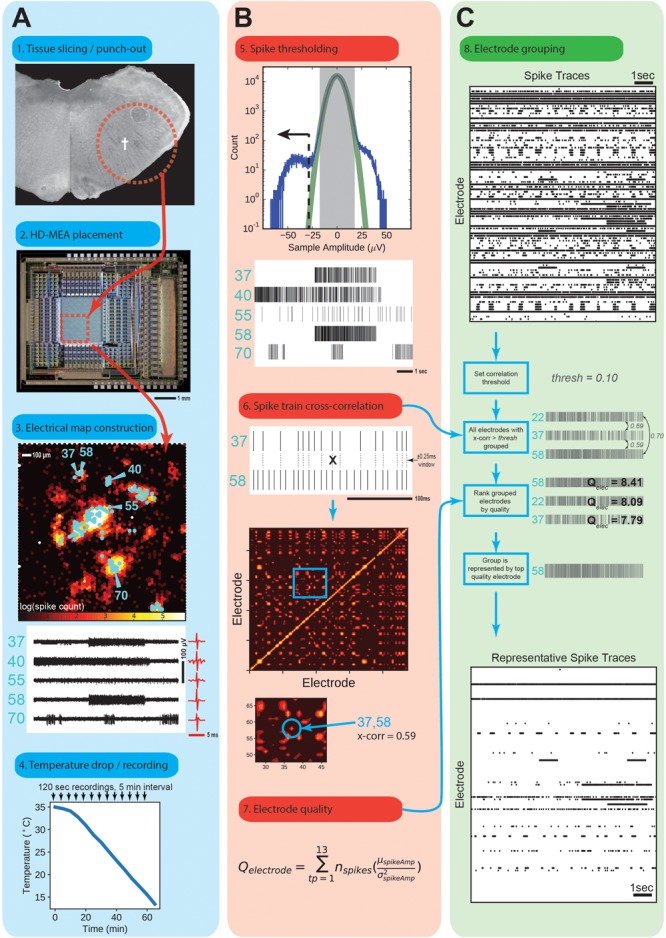
Spike Detection and Sorting. **(A)** Acute slice recordings. The tissue punch **(1)** was anchored onto the HD-MEA array surface **(2)**, central red dotted square) with an adjustable membrane. **(3)** Spike rate map of the area inscribed by the red box from above. Scale is the log of the number of spikes over a 120 s recording. Electrodes for recording were selected manually (indicated in cyan) around areas of obviously high spike rates. Five example electrodes were numbered next to their raw traces for further illustration. **(4)** Temperature drop experiment time-course. Sample in temperature is dropped from approximately 35°C to 14°C over the course of 1 h, and recorded for 120 s in 5 m intervals. **(B)** Thresholding of raw traces to obtain spike times. **(5)** A Gaussian (green line) is fitted to the center-most three standard deviations of raw recording sample amplitudes from each electrode, and extrapolated. A threshold was chosen based upon a sample value that would give a 1 in 1 × 10^6^ false positives according to the fitted Gaussian. Every sample below this is marked as a spike peak to form a binary spike trace. **(6)** Binary traces of all recording electrodes were cross-correlated within a 0.25 ms window to obtain a cross-correlation map. **(7)** Electrode quality was measured by the indicated formula, which reward total number of spikes and higher mean amplitudes, and penalizes a large standard deviation in those amplitudes. Quality was measured as the sum over all temperature points for that electrode. **(C)** Grouping process for selecting a representative electrode for unique spike traces. Binary spike traces **(8)** were grouped together if their cross-correlation was above a set threshold (0.1). The highest quality electrode in that group was selected as a representative spike trace.

After decapitation, brains were subjected to 3 min of global hypoxia before being rapidly chilled to 4°C and sliced in cutting solution designed to silence electrical activity (see “cutting solution” above). After separating the 1.2 mm diameter around the ventral respiratory column (VRC) with a circular punch (2–4 punches per animal), they were immediately submerged in 34°C recovery solution, where electrical activity was allowed to resume and recover. During the following 1 h recovery, labels for dead-live (PI and DAPI), calcium (Calcium Green), and oxidative stress (CellRox) were added. Images of tissue punches were taken and pixel intensity was measured for a central square in the tissue. Punches were then digested in an ATP assay kit for 30 min and recorded on a luminescence plate reader. Measured values for individual punches were collected in empirical distribution functions for each assay, in Figure 2B–E.

### Image Analysis

Color channels from punch images were separated with Nikon Elements Viewer and combined into a stack in ImageJ (National Institutes of Health, Bethesda, Maryland, United states). A single central box was drawn, carefully avoiding the edges, and total pixel intensity for each channel was recorded.

### High-Density Microelectrode Array

We employed a complementary metal-oxide semiconductor (CMOS)-based high-density microelectrode array (HD-MEA) for recording extracellular field potentials from acute brain slices (Frey et al., 2010). The system features 11,011 metal electrodes, 126 of which can be simultaneously recorded from. Electrodes were 7 μm in diameter and spaced 18 μm apart, yielding a density of 3150 electrodes per mm^2^. Electrodes were electrochemically coated with platinum black, which resulted in a baseline noise level of approximately 3–4 μV_rms_. Recordings were performed with a final gain of 1450x, sampled with 8 bit resolution (at 8 μV/bit) at 20 kHz sampling rate and stored uncompressed on hard disk for further processing (see below).

### Electrophysiological Recordings

Tissue punches were placed on top of the HD-MEA with the underside touching the electrodes. The punch was then held down with a custom alignment cone with a nylon mesh. Recordings were performed under constant perfusion (0.5 ml/min) with oxycarbon (95% O_2_, 5% CO_2_)-gassed recording solution (same as recovery, but with 9 mM KCl). The HD-MEA was placed on a temperature-controlled plate (Teca Corp., Chicago, IL, United States) which was also used to control the temperature of the solution by running perfusion tubes over it. For each punch the spontaneous activity was first mapped by performing a scan at 35°C of all 11,011 electrodes, 125 at a time, in random sequence. Up to 125 electrodes were then selected for subsequent recordings in the areas of high spike rates (Figure 2A). Nine animals were used for each adaptation condition (autumn or winter), and a single punch was used per animal. For each punch between 13 and 88 electrode locations were selected for a total of 262 and 348 unique electrodes from the autumn control group and winter hibernation-prepared group, respectively, for subsequent analysis. Over the course of 1 h the temperature was continuously dropped from 35°C to 14°C. A temperature drop of approximately 21°C was confirmed by the HD-MEA’s on-board temperature sensor. Recordings were performed for 120 s every 5 min, yielding 13 temperature points per experiment.

### Electrophysiology Data Analysis

Raw voltage traces were filtered (300–5000 Hz bandpass) using a custom Matlab script to produce filtered traces (Figure 2B,C). Spike events were found using a custom software script programmed in an IPython Jupyter notebook. Briefly, for each electrode and temperature point, all sample amplitudes were binned relative to their mean (close to zero). A Gaussian distribution was fitted to the samples within ± 3 standard deviations, which were assumed to be noise. A voltage amplitude threshold of five standard deviations was then used, giving a false positive detection rate of one in 1 × 10^6^ samples, which corresponds to a false-positive spike rate of 0.02 Hz. Electrodes that did not show an average spike rate of at least 0.25 Hz during at least one temperature point recording were discarded. Electrodes, where more than 50% of spikes appeared at the same time were grouped together into a single electrode family. These electrodes were assumed to record from the same neuron. To prevent over-representation of correlated activity detected on multiple electrodes, only one electrode for each family was kept. This selection of “unique electrodes” yielded non-overlapping (maximally distinct) spike patterns.

We characterized the complexity of the spike trains by the coefficient of variation (CoV = SD/Mean) of the inter-spike intervals (ISI). The ISI is the time lag between subsequent spikes on a single electrode. A CoV = 1 refers to random spike train representing a Poisson process with an exponential distribution of ISIs, whereas tonic (periodic) and bursting activity yields a CoV below or above 1, respectively.

### RNA Extraction

For RNA sequencing, brains were rapidly removed and snap-frozen in 2-methylbutane (Sigma-Aldrich, Buchs, Switzerland) cooled to -40°C, and stored at -80°C until slicing. Prior to slicing, brains were allowed to equilibrate to -20°C for at least 2 h, then sliced in 25 μm coronal sections on a cryostat. Slices were placed onto laser-dissection slides (Molecular Machines & Instruments, Eching, Germany), fixed with pure ethanol and rapidly air-dried. A box area around the WGA-Alexa488 labeled pre-Bötzinger was laser dissected. Both contralateral nuclei of three slices were included per tube. This was repeated over a series of 10 sample tubes in order to collect cells through the entire labeled region. Samples were placed on dry ice and stored at -80°C for later RNA prepping. Total RNA was extracted from each tube using a total RNA extraction kit (Norgen Biotek, Thorold, Canada).

### RNA Sequencing

RNA was pooled from the 10 sample tubes, giving a single RNA sample per animal. Three and two replicates for each adaptation group (autumn and winter) were used, respectively. Libraries were prepared using an Illumina non-stranded TruSeq mRNA kit. The libraries were then run on an Illumina NextSeq 500 using a high output 75 cycle kit, producing 26–38 million reads per replicate (total reads: 67.4 M and 115.3 M for winter and autumn, respectively).

### RNASeq Analysis

Reads were aligned to the *Mesocricetus auratus* 1.0 genome (NCBI genome ID 11998) using TopHat 2.0.13 (Cossio et al., 2012)/Bowtie 2.2.5 (Langmead et al., 2009). Reads were then counted per exon using htseq-count (Anders et al., 2014), the output of which was then used in the FactoMineR package for R (Lê et al., 2008) for quality analysis. Reads were then used in the SeqGSEA package for R (Wang and Cairns, 2014) to obtain a set of significantly changed genes. Separate differential expression (DE) and differential splicing (DS) indices were generated, and subsequently combined using the “rank” method with a DE/DS weighting of 0.25/0.75 to produce an integrated gene score for each gene. Scores were randomly permutated 1,000 times in order to estimate *p*-values for each enrichment score. Genes with a *p*-value of < 0.001 (950 total) were used for further gene ontology (GO) analysis.

### Gene Set Enrichment

We performed enrichment analysis of GO terms associated with our gene list by first generating 100,000 permutations of 950 randomly selected gene names from the hamster genome (without replacement), and accumulating counts of how many times each GO term appeared per permutation. This produced a 100,000-sample random distribution of counts for each GO term. We compared the GO terms produced by our gene list to these distributions and accepted terms which had the following criteria: (1) a *p*-value of less than 0.05 according to its permutation distribution, (2) a count of at least 4 (a particular term is referenced by at least 4 genes), and (3) were terminal (no other terms produced by our list referenced this as a “parent” term).

### Splice Junction Analysis

Tophat-generated bam files were processed by the QoRTs suite (Hartley and Mullikin, 2015b) to perform junction count analysis. Results were further processed for differential usage of exons and visualized by the JunctionSeqR package for R (Hartley and Mullikin, 2015a).

## Results

### Respiratory Neurons Are Recorded by Microelectrode Array

We were able to target medullary neurons using retrograde synaptic tracing of a WGA injection into the pleural cavity (Buttry and Goshgarian, 2014, 2015). The area of the medulla that was labeled by WGA is slightly caudal to the NA, and appears to be anatomically co-spatial with the location of inspiratory pre-motor neurons according to (Funk and Greer, 2013). This location is superior to the preBötC in other mammals (Figure 1D). To confirm this in hamster, WGA-labeled hamster brainstems were sliced in 100 μm-thick free-floating sections, and first checked for labeling (Figure 1Ea), followed by confirmation of landmarks associated with the correct coronal slice depth of the preBötC (Ruangkittisakul et al., 2014), such as the presence of a fourth ventricle [Figure 1Ea (^∗^)], a prominently layered inferior olive [Figure 1Ea (‡)], and a pre-compact, diffuse NA [Figure 1Ea (†)]. We then stained the slices using an anti-somatostatin antibody. Images revealed diffuse somatostatin labeling cospatially with WGA labeling (Figure 1Eb–d). Upon counting normalized pixel intensity along a dorsal-ventral linear profile (Figure 1Ed, arrow), the majority of somatostatin staining was found to be cospatial or ventral to the WGA-labeled region. The presence of a second peak in somatostatin intensity (Figure 1Ee, §) suggested the preBötC is situated at this location as in other rodents (Koizumi et al., 2016).

Three-hundred micron thick slices taken at a similar coronal slice depth (as judged by landmarks and WGA labeling) were punched by a circular knife to an area of 1.8 mm diameter around the WGA-labeling for further analysis (Figure 2A). Punches were scanned for electrogenic activity and associated signal waveforms using a CMOS-based HD-MEA (Frey et al., 2010). The system featured 11,011 metal electrodes, 126 of which can be simultaneously recorded from. Electrodes were 7 μm in diameter and spaced 18 μm pitch, yielding a density of 3150 electrodes per mm^2^. This platform was used to broadly measure cellular viability in the ventral respiratory column (VRC) of the medulla, and more specifically, circuit functionality of a subset of the VRC, the preBötC.

Up to 126 electrodes were selected in areas of high activity (Figure 2A3), and signals for these electrodes were recorded over 120 s. Figure 2A3 (below) shows typical recordings from this region, some of which displayed complex, bursting action potential patterns of intervals from 2 to 15 s. WGA labeling, somatostatin staining, anatomical landmarks, along with electrophysiological recordings support the case that we can target the hamster preBötC for analysis.

### Electrophysiological Function Is More Robust in Winter-Adapted Animals

We wanted to investigate how robust respiratory circuitry performed over a range of temperatures that would be seen by an animal entering a hibernation bout. We obtained tissue punches having the same location and diameter as in the previous experiment, but of 300 μm thickness. Animals from each adaptation group (*n* = 10) were used, from which a single punch was recorded from. After recovery from preparation, we recorded action potentials on tissue punches on a HD-MEA, selected electrodes for further recording in areas of high spike activity, and consolidated electrodes that gave similar spiking patterns (see section “Materials and Methods”). We obtained a total of 262 and 348 electrodes for the autumn and winter groups, respectively. Raw spike traces were then thresholded to obtain spike peak times, spike time cross-correlations, and electrode quality measures (Figure 2B). Electrodes were then grouped and eliminated according to Figure 2C. Each of these grouped units represented an electrode with a unique spike pattern.

**FIGURE 3:**
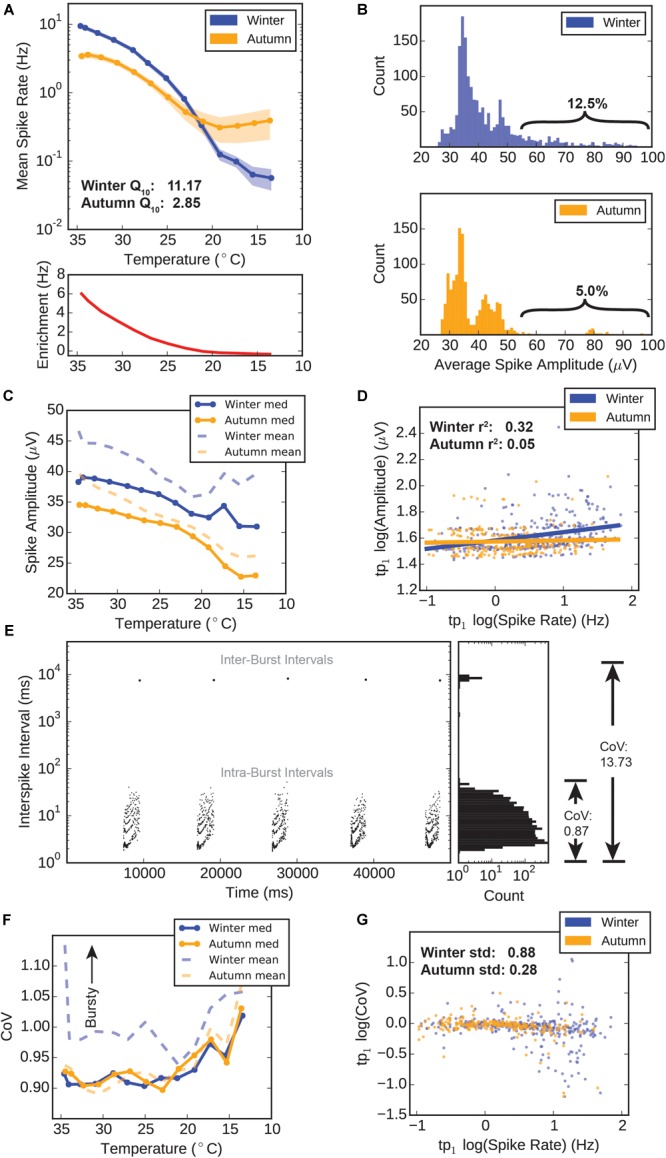
Winter versus autumn HD-MEA electrophysiology. **(A)** Spike rates versus temperature. Top: Mean spike rate recorded from electrodes during temperature drop. Error ± standard error of the mean (SEM). Bottom: “Winter Enrichment” factor (red) calculated by subtracting the average autumn spike rate for each temperature point from the corresponding average winter spike rate for each temperature point. **(B)** Distributions of electrode spike amplitudes. The amplitudes for all spikes recorded at three temperature points (tp_0_–tp_2_, 34.6–32.7°C) were averaged, thus giving a single average amplitude value for each electrode. These values were counted in 1 μV bins for the winter group (top) and autumn group (bottom). **(C)** Electrode spike amplitudes during temperature drop. The amplitudes for all spikes recorded at any given temperature point were averaged to a single value for each electrode. The median (solid lines) and mean (dashed lines) values for all electrodes are plotted. **(D)** Scatter plot and linear regression of amplitudes versus spike rates at tp_1_ (34.1°C). Log spike amplitudes were obtained by averaging all spike amplitudes recorded on a particular electrode, and calculating the log_10_, yielding a single value for each electrode. Log spike rates were obtained by dividing the total spike count for each electrode by the recording time (120 s), and calculating the log_10_. Solid lines represent a linear regression for these values within each group. **(E)** Example calculation of spike complexity (coefficient of variance). Plot shows data from a bursting spike train, with spikes scattered in time versus inter-spike interval (ISI) (left), and the distribution of interspike intervals (ISI) (right). A non-bursting train would only include ISI and have a relatively low CoV of 0.87. When inter-burst intervals are included, CoV jumps to a high value (13.73), indicating that CoV is a good measure of how “bursty” or “complex” spike trains. Poisson-distributed trains would have a CoV value of 1. **(F)** Electrode coefficient of variance during temperature drop. A single CoV is derived for each electrode according to the methods. The median (solid lines) and mean (dashed lines) values for all electrodes are plotted. **(G)** Scatter plot and linear regression of coefficient of variance versus spike rates at tp_1_ (34.1°C). Log CoV and log spike rates from each electrode are averaged to a single value for each electrode.

We recorded spike times and amplitudes over a range of temperatures from euthermic (∼35°C) down to what would be a deep cooling bout in a hibernating animal (∼14°C), at 5 min intervals over a 60 min drop (Figure 2A4; 13 temperature points spaced approximately 1.66°C). At euthermic conditions winter and autumn groups showed initial spike rates of 9.43 ± 0.68 and 3.42 ± 0.41 Hz (Mean ± SEM), which were significantly different (M-W test, *p* = 5.20 × 10^-7^). The winter group retained this higher spike rate until approximately 23°C, below which spike rates between the two groups converged and became indistinguishable (Figure 3A, blue and orange lines; note the logarithmic *y*-axis scale). The difference in spike rate between the winter and autumn group (Figure 3A, red line) is highest at euthermic temperatures, and rapidly drops initially, followed by a slow approach to 0 Hz, as spike rates between the two groups also tend toward zero. The calculated full-range Q_10_ temperature coefficient, a measure of temperature sensitivity for biological systems, was 11.17 for the winter group, which far exceeds that of the autumn group (2.85), indicating a higher level of spike rate temperature sensitivity in the winter group.

We took advantage of the HD-MEA’s ability to record action potential spike waveforms in addition to exact spike times, in order to see if seasonal adaptation modified this aspect of action potentials in the respiratory center. Amplitude distributions in the first three temperature points (tp_0_–tp_2_) (approx. 34.6–32.5°C) show distinctly different shapes for each group. Winter amplitude counts peak near 35 μV and exhibit an apparent exponential decay in count as amplitude values increase (Figure 3B top). The autumn distribution is distinctly bimodal, with peaks at 35 and 42 μV, and a separating trough at 40 μV (Figure 3B bottom). Fewer electrodes populate the 55 μV and higher region (braces) compared to the winter group (5.0 versus 12.5%), showing that the autumn preBötC contains fewer neurons that are capable of moving large amounts of charge during an action potential.

Throughout the hypothermic temperature range, winter means and medians of spike amplitudes remain higher than autumn means, and even diverge further below 20°C (Figure 3C). Amplitudes at all temperature points are significantly different between the groups (M-W test, *p* < 0.05). We observed a weak linear correlation (*r^2^*= 0.32) in the winter group between log amplitude and log spike rate values at tp_1_ (34.1°C), whereas this relationship is almost non-existent in the autumn group (*r^2^*= 0.05) (Figure 3D). This suggests that the winter group possesses a subset of neurons which are simultaneously high rate and amplitude, which seems absent in the autumn group.

The third parameter we calculated for the purpose of assessing circuit performance was the CoV of the spike trains. Briefly, this measure is calculated by dividing the standard deviation of an electrode’s ISI by the mean spike rate. This measurement is naturally higher for bursting spike trains due to the large periodic inter-burst interval that dramatically increases the standard deviation of the ISI, as demonstrated in Figure 3E. Therefore, we use this metric to identify highly ordered, bursting spike train signals.

The median CoV throughout the near-hypothermic temperature range (35–20°C) for both groups remained near 0.90, increasing to approximately 1.00 below 20°C (Figure 3F). Most interestingly, however, was the measured value for the mean CoVs: with falling temperature, the mean CoV for the winter group remained substantially higher than its median CoV. By comparison, the autumn group’s mean CoV remained near its median CoV. This shows that the distribution of CoV values in the winter group has an upward skewness, that is, there exist a small number of spike trains which are highly “bursty.” When looking at a scatter plot of log CoV versus log spike rate, we can see that there is more variability in CoV values at higher spike rates in the winter group, whereas the values of the autumn group were fairly uniform at all spike rates (Figure 3G). In fact, the standard deviations of CoV values were 0.88 and 0.28 for winter and autumn groups, respectively.

These results suggest that there are substantial differences in the electrophysiological behavior of the autumn and winter group respiratory centers. In particular, the winter group possesses neurons that have higher spike rates, higher amplitudes, and a higher degree of diversity in terms of spike train complexity: there are more neurons sustaining bursts as well as neurons that spike randomly. This is likely explained by a seasonally-induced adjustment of various electrophysiological characteristics of the respiratory circuit in the winter group.

### Winter-Adapted Neurons Display Altered Action Potential Waveforms

**FIGURE 4:**
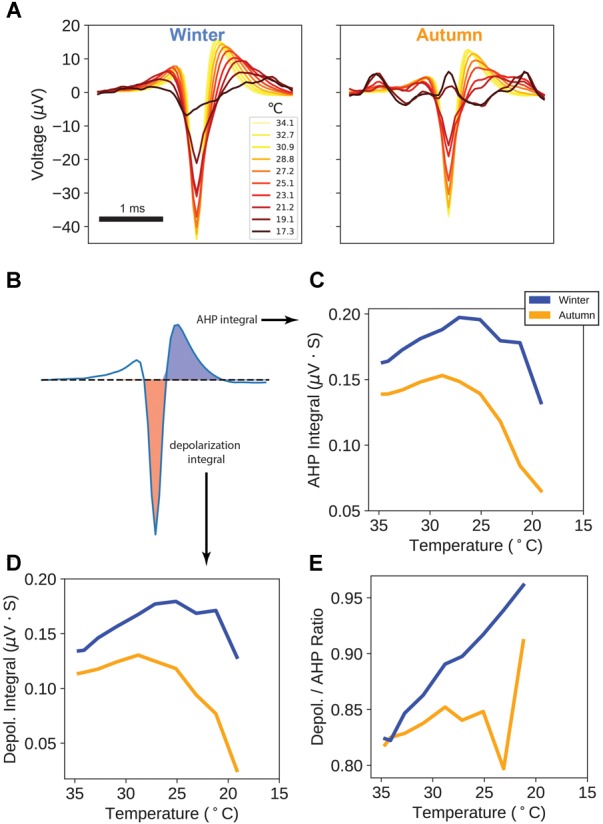
Action Potential Waveforms. **(A)** Average action potential waveforms taken from a sampling of every 20th action potential on each unique electrode, for each temperature point. All measurement units (μV) are as measured at the MEA electrode. **(B)** Areas used in calculation of after-hyperpolarization (AHP) and depolarization integrals. **(C)** AHP integrals versus temperature. **(D)** Depolarization integrals versus temperature. **(E)** Ratio of Depolarization over AHP integrals versus temperature.

We took further advantage of the availability of raw recording traces and spike peak locations to investigate how action potential waveforms behave over temperature drops. Figure 4A shows the average action potential waveforms for every 20th detected action potential for the first ten temperature steps, up to the point of apparent failure. Even at 17.3°C, spike peaks for winter samples are apparent, whereas autumn sample peaks endure only down to 21.1°C, below which recordings become noise.

For each temperature point, we measured the action potential’s depolarization integral (area under the curve) and after-hyperpolarization (AHP) integral, as a proxy measure for electrical charge transfer (Figure 4B). Interestingly, both AHP and depolarization integrals in the winter samples display an increase in magnitude down through 25°C (Figure 4C,D), with autumn samples showing a flatter trajectory with an earlier peak and steeper drop-offs. Taking the ratio of depolarization integral over AHP integral shows a curious property: winter sample ratios increase throughout the temperature drop whereas autumn sample ratios remain flat up until the point of action potential failure.

These findings suggest that after seasonal adaptation, action potential waveforms are being actively modified, likely by a change in ion channel or pump kinetics, for the purpose of sustaining larger, longer signals.

### Seasonal Adaptation Reduces Cell Death but Not Enough to Affect ATP Production

**FIGURE 5:**
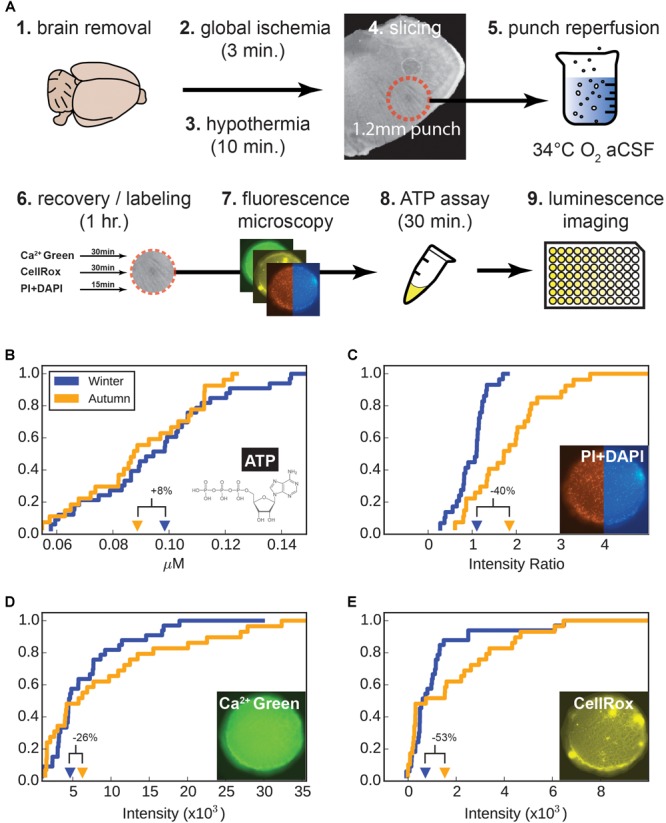
Viability assays. **(A)** Tissue preparation, hypoxia-reperfusion of tissue and subsequent processing. **(B–E)** Empirical cumulative distributions for tissue punch measurements. For each parameter measure of viability (ATP, cell death, calcium, and oxidative stress), values step-up by 1/n (for *n* tissue punches) at their corresponding value on the *x*-axis. The distributions are as follows: **(B)** Adenosine triphosphate (ATP), **(C)** cellular dead-live ratio (PI/DAPI), **(D)** Ca^2+^ (Calcium Green), and **(E)** CellRox (oxidative stress), respectively. Percent changes indicate winter values in relation to autumn values. Insert images show a representative tissue punch at 10× magnifications.

In addition to seasonally-induced genetic changes causing a shift in the electrophysiological characteristics of the respiratory circuit, it is also possible that circuit properties are being altered by the tissue preparation. Presumably if part of the winter-adaptation process is the induction of elevated neuroprotection in the animal’s brain, a larger portion of the neurons in non-adapted circuits could be killed off when the brain was removed and processed. Therefore we sought to test the relative viability of brain tissue samples in both groups by performing a panel of viability assays that measured absolute levels of ATP, relative slice levels of cell death, calcium accumulation, and oxidative stress (Figure 5A).

Adenosine triphosphate is accepted as a good indicator of when cells are damaged and will likely undergo cell death in the near future; lower levels of ATP indicate unhealthy cells. The second assay measured the ratio of dead to live cells, which is a snapshot of the relative number of cells which have been killed already. The third measured intracellular calcium accumulation, an indicator of neuronal stress, particularly over-excitation. The final assay measured oxidative stress, particularly the accumulation of reactive oxygen species (ROS), which accumulate if cells are subjected to hypoxia and reperfusion, and don’t have effective means to clear ROS.

Nine animals were used from each adaptation group (autumn and winter), from which 2 to 4 punches were obtained and measured for each of the four viability assays. There were 29 and 32 samples for the autumn and winter groups, respectively. For each assay class we plotted individual values for all tissue punches (Figure 5B–E).

Since each distribution cannot be assumed to be normal, we performed a Mann-Whitney U (M-W) test to test the rank of samples for each adaptation group (Table 1A). ATP, calcium, and oxidative stress were found to be not different for autumn and winter samples (*p* = 0.252, 0.353, and 0.301, respectively), but the rank of dead-live samples (the ratio of PI to DAPI fluorescence intensity, Figure 5C) showed an unequivocal difference between the autumn and winter groups in terms of rank (*p* = 2.22 × 10^-5^), with the winter group having a 48% lower mean than the autumn mean.

**Table 1:** Statistical tests for viability assays.

(A) Mann-Whitney U Test	
**H_0_ = samples have no rank difference**	
ATP:	0.252
live/dead:	2.223 × 10^-5^
Ca^2+^:	0.352
Rox:	0.301
**(B) Kolmogorov-Smirnov 2-sample**	
**H_0_ = samples come from same distribution**	
ATP:	0.836
live/dead:	3.374 × 10^-5^
Ca^2+^_:_	0.508
Rox:	0.023

*Test names and null hypotheses (H_*0*_) indicated in header. Sample groups highlighted in green indicate those failing to reject H_*0*_.*

We also performed a Kolmogorov-Smirnov 2-sample test (K-S test, Table 1B), which tests if two distributions are different from each other. As expected, the dead-live distribution were different between autumn and winter (*p* = 3.37×10^-5^), and ATP and calcium showed no difference (*p* = 0.836 and 0.508, respectively). Interestingly, the oxidative stress distributions (Figure 5E) did show a difference according to the K-S test (*p* = 0.0231). This difference was likely driven by the apparent lack of mid-level intensity counts for the winter group (Figure 5E). A 30% lower winter mean value in conjunction with the altered value distribution suggested that more cells were under a higher level of oxidative stress in the autumn group, although high sample scatter prevented this from being statistically significant.

Results from our viability assays indicate that while respiratory centers in winter group animals were susceptible to decreased levels of hypoxia/reperfusion-induced cell death and oxidative stress, absolute numbers of viable cells, as indicated by nearly similar ATP levels, were unchanged. What this likely indicates is that the increase in cell death seen in the autumn group is significantly elevated in comparison to an already low level of cell death in the winter group. This higher level of death is, however, not significant enough to show a measurably lower level of ATP in autumn group (see section “Discussion”).

### RNASeq Reveals 950 Genes With Differential Expression and Splicing

**FIGURE 6:**
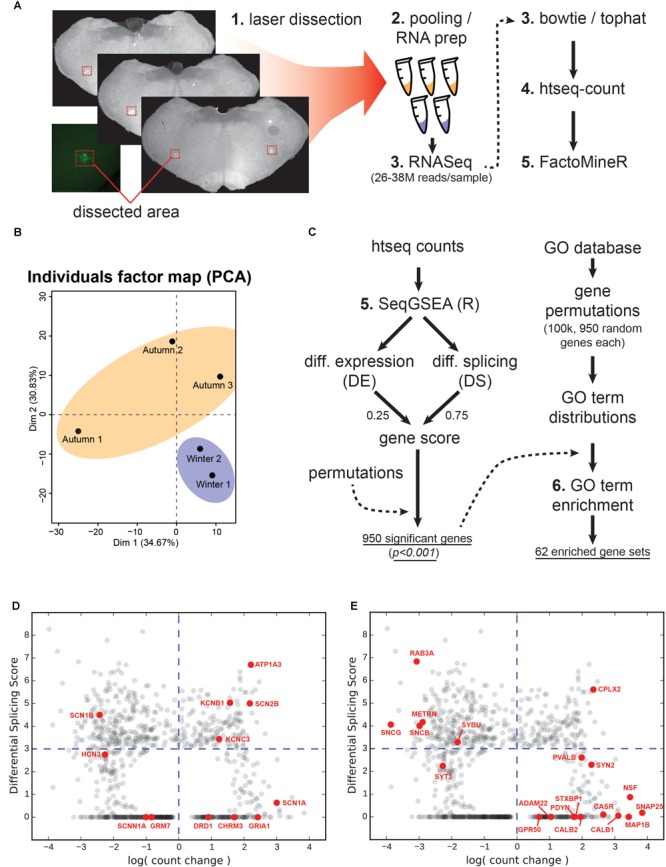
Tissue collection, RNA sequencing and analysis. **(A)** Laser dissection, RNA and library prep, RNA sequencing preparation suite. **(B)** Principal component analysis showing two dimensions for the autumn versus winter samples. **(C)** Work flow for gene set enrichment. Left-hand steps produced the total number of significantly-changed genes based upon a combined differential expression (DE) and differential splicing (DS) gene score as produced by the SeqGSEA package for the R programming language. Right-hand steps use a Gene Ontology (GO) term database to produce a distribution for each GO term based on 950 randomly-selected genes. The actual significant genes from the left-hand steps were then used to generate GO terms, which were compared against the permutated distributions to test for significance. **(D)** Count change versus DS for ion handling genes. Genes encoding ion channels, metabotropic receptors, and pumps are highlighted in red. Grey points indicate all 950 genes from the screen. Blue dashed lines represent neutral count change (vertical; zero log-fold-change) and the minimum DS score for significance (horizontal). **(E)** Count change versus DS for structural genes. Genes that serve either a structural function, support or regulate structural elements are highlighted in red.

We targeted WGA-Alexa488 labeled regions for RNA sequencing by using laser dissection, subsequent RNA extraction and preparation as illustrated in Figure 6A. Each of the 3 autumn and 2 winter samples yielded 26–38 million reads, which were then processed using the bowtie/tophat suite, followed by htseq-count. The FactoMineR package for *R* was used to generate a principal component analysis for all samples (Figure 6B), which revealed separation of the two groups.

### Gene Set Enrichment Implicates 62 Significant Gene Ontology Processes

We then processed the counts using the SeqGSEA package for *R* (Figure 6C), which generated a weighted gene score based on Differential Expression (DE) and Differential Splicing (DS). Both quantities by definition are always positive, with DE representing the magnitude of change in a particular gene between experimental (winter) and control (autumn) samples. We generated 1000 permutations by SeqGSEA in order to empirically determine *p*-values for each DE and DS score. For a combined gene score, we blended DE and DS with 0.25 and 0.75, respectively. This generated 950 genes with a significantly different gene score (*p* < 0.001).

In order to determine which genetic processes were enriched by the DE and splicing of these genes, we created our own non-parametric statistical test using the MouseMine ^1^ GO database. This service returns a set of standardized GO terms that a given gene is associated with. Each term is situated within a descriptive hierarchy of other terms that describe a biological process. However, a set of several hundred genes will implicate several thousand GO terms, and we wanted to know if the particular combination of genes produced by our screen implicated certain processes *more* than a random selection of genes. We therefore needed a reference for the background occurrence of any given GO term to compare against. This reference was created by randomly selecting 950 annotated gene names from the hamster genome, querying the MouseMine database, and counting the number of times each GO term was referenced. This permutative process was repeated 100,000 times, the results of which we used to build a distribution of occurrences for each GO term. We then queried each gene name in our list of significant genes to get a complete set of associated GO terms. Any term whose occurrence was in the top 5% of its randomly permutated distribution, in addition to occurring at least five times, was marked as significant. This process generated 62 GO terms (Figure 6C). Any given GO term resides on a hierarchy of descriptiveness, up to nine levels deep, shown in Supplementary Table S1.

## Discussion

In this study, we directed hamsters to enter a hibernation-preparatory state by using day-length cues. This manipulation naturally causes Syrian hamsters to enter a physiological state that is required for hibernation to occur, and allowed us to compare this “winter” state against an unprepared “autumn” state. Our electrophysiological data show that respiratory circuitry changes in terms of spike rates, amplitudes, and complexity, in a way that suggests an increase in low-temperature robustness. Although spike rates converge and the autumn group sustains its rate better at low temperatures (Figure 3A), the difference in spike rate is less than 0.5 Hz in aggregate, whose *in vivo* significance is difficult to interpret in an acute slice culture where many of the excitatory inputs to the rhythm generator are severed. Through most of the temperature drop the winter group spike rate remains higher than the autumn group’s rate, indicating that the winter-adapted respiratory center is “primed” for higher electrophysiological activity at euthermia.

Additionally, winter-group action potentials show more robust kinetics in terms of depolarization and AHP integral as a stand-in for electrical charge transfer by having higher magnitudes (Figure 4C,D). We found the additional curious property that the depolarization-to-AHP ratio climbs in the winter-group as temperature drops (Figure 4E). These electrophysiological results suggest the presence of active seasonal mechanisms to bolster circuit connectivity and alter sodium and potassium channel charge transfer.

Our RNA sequencing data supports these possibilities. We isolated 950 genes that had significantly different gene scores between the groups. This is a strikingly similar number to a previous study that investigated seasonal differences in ground squirrel hypothalamus (Schwartz et al., 2013). Through gene set enrichment analysis, we produced 62 uniquely enriched GO terms (Supplementary Table S1), each of which described a biological processes associated with specific sets of genes. We also took a more conventional approach to gene enrichment analysis by ranking according to expression, splicing, fold-change, and absolute count change (Supplementary Figure S1). By taking the highest (lowest) ranking members of these categories, we were able to produce additional relevant genes associated with hibernation changes. The following discussion outlines two major genetic processes and supporting genes we uncovered.

### Ion Channels and Pumps

In level 7 of the GO term hierarchy (Supplementary Table S1) we find a term related to sodium channel activity (GO:0005272). More specifically, the winter group showed downregulation of three ion-handling proteins (*Hcn3, Scn1b*, and *Scnn1a*) and upregulation of an additional three (*Scn2b, Scn1a, Kcnc3*) (Figure 6C). *Hcn3* is a hyperpolarization activated cyclic nucleotide gated potassium channel, which belongs to a category of channels responsible for pacemaking currents (Robinson and Siegelbaum, 2003; Biel et al., 2009). *Hcn3* has also been found to be essential for generating rhythms in the intergeniculate leaflet (Ying et al., 2011). In our screen this gene was found in the top DE category and was the only member of this GO term in that ranking, and its downregulation and moderate resplicing in the VRG likely indicates an adjustment to some component of the breathing rhythm transmission circuitry, and could explain the difference in spike statistics seen between the two groups. *Scn1b* is a voltage-gated sodium channel subunit, which is essential for controlling the inactivation of sodium channels (Brackenbury and Isom, 2011), and is known to interact with both voltage-gated sodium and potassium channels, the latter via its voltage sensing and pore domains (Nguyen et al., 2012). It should be noted that *Scn1b*’s significance in our screen is primarily due to its DS. Although the literature is scant on the remaining ion channel subunits, their presence is evidence of an apparent swapping of subunit composition and suggests that neurons are fine-tuning the kinetics of action potential propagation. These six genes are likely candidates for explaining the altered depolarization and AHP kinetics seen in Figure 4. It has been suggested that conduction amplitude and velocity at low temperatures are dependent upon action potential rise time, which is largely governed by sodium channel kinetics (Baylor and Stecker, 2009).

**FIGURE 7:**
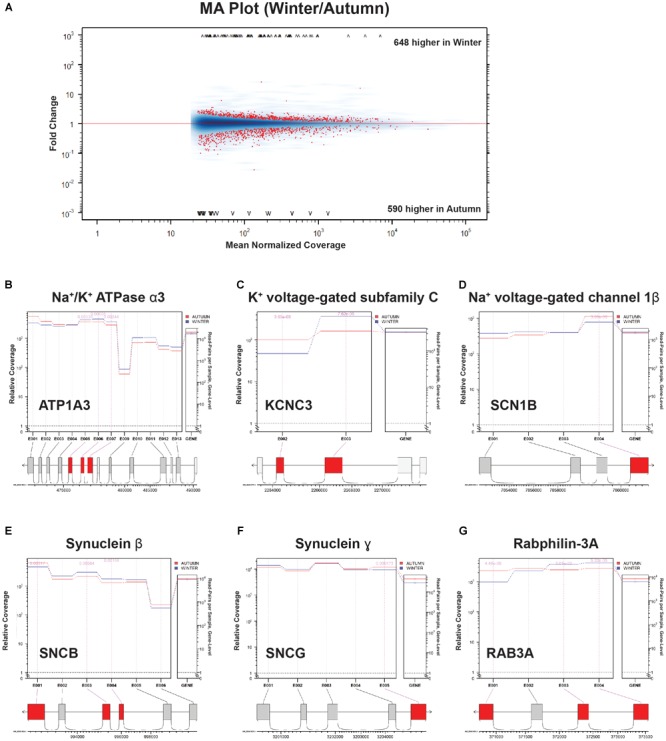
Splice junction analysis. **(A)** MA Plot Winter versus Autumn. Fold change (“M,” *y*-axis) versus Mean Normalized Coverage (“A,” *x*-axis) for all detected genes. Significant genes (*p* < 0.01) highlighted in red. **(B–G)** Coverage/Expression profile plots. Exons that show significant DE (*p* < 0.01) are highlighted in red.

*Atp1a3* is a crucial subunit to an ATP-driven ion pump that is essential for maintaining Na^+^ and K^+^ gradients across the membranes of neurons (Ikeda et al., 2004), and appears in several mid-hierarchy GO terms (Supplementary Table S1, levels 3 and 4). Heterozygous mutations in this gene are implicated in a variety of neuronal disorders that share a collection of symptoms such as seizures, hypotonia, ataxia, chorea, and paralysis (Sweney et al., 2015). Interestingly, a recent study has uncovered that *Atp1a3^-/-^* knockouts could not maintain rhythmic inspiratory bursts for more than several hours compared to heterozygous and wild-type controls, and showed lowered basal bursting rates (Ikeda et al., 2017), implying impairment in the respiratory circuitry. Ikeda et al. (2017) also confirmed the expression of *Atp1a3* at the NA, near where our samples were taken. *Atp1a3* showed not only a high positive count change, but also had a highly ranked DS score (Figure 6D), which we confirmed with junction analysis (Figure 7B). This suggests that this upregulated gene is also changing out part of its protein structure, possibly to a sequence having resistance to low-temperature or energy-starved conditions.

### Neuronal and Synaptic Structure

Supporting the notion that the respiratory center is undergoing restructuring, the highest two positive count changes of any gene occurred for *Snap25* and *Nsf* (Figure 6E). Snap25 is a SNARE-binding protein crucial for vesicle release at synaptic terminals (Rizo and Xu, 2015), and Nsf works in conjunction with Snap25 for rapid disassembly of SNARE complexes (Rizo and Xu, 2015; Ryu et al., 2016). The high level of upregulation of these two would suggest either an expansion in the number of synapses or an enhancement of the release machinery in currently existing synapses. In either case, an increase in signal-transmission capacity during the winter season is implied. Another crucial SNARE-binding protein, *Cplx2*, also undergoes a high degree of upregulation and DS. This protein facilitates vesicle fusion by “amplifying” the effect of Ca^2+^ influx and is, therefore, a positive factor in vesicle release (Trimbuch and Rosenmund, 2016). It should be noted that in addition to these three noteworthy genes, our screen included four GO terms pertaining to the axon terminal: “SNARE binding,” “Syntaxin-1 binding,” “axon terminus,” and “terminal bouton” (GO:0000149, GO:0017075, GO:0043679, GO:0043195), suggesting that this structural element and its release machinery is under intense regulation.

Structural manipulation is not limited to the synaptic terminal, however. The third highest increase in count change occurred in *Map1b*, which produces a microtubule-associated protein heavily involved in axon regeneration and neurite branching via the control of underlying microtubule dynamics (Barnat et al., 2016). Perhaps closely related to this same process is *Casr*, a gene whose protein promotes axon and neurite extension in response to external calcium. This extension is most pronounced during developmental stages where axons are ramifying and branching at their distal targets, as opposed to the extension of said axons to their targets (Vizard et al., 2008, 2015). Both *Mapb1* and *Casr* upregulation, in conjunction with synaptic terminal machinery upregulation perhaps gives a clue about what’s happening in the axon: neurite outgrowth and an increase in synaptic contacts but not *de novo* extension of long-range connections. Figure 6E summarizes DS versus count changes.

### Circuit Restructuring Versus Dysfunction

There is, however, an alternate explanation for the increased robustness in winter-group circuit function. First, higher rates of cell death in the autumn group could be eliminating enough neurons from the circuit such that it passes below a minimum critical threshold for burst perpetuation. This follows the “group-pacemaker hypothesis,” whereby respiratory bursts are thought to be an emergent property of recurrent excitatory synaptic connections and can be generated in the absence of autonomous pacemaker neurons. Individual pacemaker neurons still exist, however, and seem to provide the required excitation for the initiation of bursts (Feldman and Del Negro, 2006). Recent progress in this area has supported the group-pacemaker hypothesis by demonstrating that progressive ablation of Dbx-1 containing preBötC neurons slows breathing frequencies, resulting in total cessation past 15% ablation of the total population (Wang et al., 2014). Thus, positive feedback excitation seems to be the model by which adult mammals generate breathing rhythms, and could be a possible explanation for respiratory failure in old age and neurodegenerative diseases (Feldman and Del Negro, 2006); progressive neuronal death eliminates excitatory contributions to the network until system failure occurs, usually during sleep. In the case of the autumn group respiratory center, a sufficient level of cell death could either be removing the requisite excitation needed to sustain bursts, or possibly too few autonomous pacemakers remain alive in the network to initiate them.

This “group pacemaker failure hypothesis,” however, is contradicted by the rest of our viability data, specifically ATP. Presumably if a critical amount of cells (>15%) were killed off during the tissue preparation, this would be evident in the measured levels of ATP in winter versus autumn groups. Our measurements showed neither a significant difference in ATP values, nor a noticeable trend thereof. Our live/dead assay cannot give absolute values of cell death and therefore suggests that while there is indeed seasonally-derived neuroprotection, it’s not enough to appreciably affect the amount of cells remaining alive and generating ATP after the tissue preparation.

### Oxidative Stress and Metabolism

It is worth briefly discussing possible explanations for the reduction in cell death in the winter group, which could be accounted for by some of the gene changes seen in our screen. We see changes in several genes involved in the response to oxidative stress (Supplementary Figure S2), in particular mitochondrial quality control at the protein and organelle level. It is, however, curious that such crucial genes would be downregulated in preparation for bouts of hypoxic oxidative stress. *Fundc1* is specifically triggered by hypoxic conditions with the purpose of removing damaged mitochondria (Wei et al., 2015). *Pink1* seems to have a similar mitochondrial quality control function for removing damaged mitochondria in conjunction with Parkin (Rüb et al., 2017). Dmpk has a function that is unclear, but it is believed that it is phosphorylated under conditions of oxidative stress and prevents the opening of the mitochondrial permeability transition pore (PTP) (Pantic et al., 2013), an action that commits cells to death. *Lonp*, which is heavily respliced in our screen, is sensitive to hypoxia and is responsible primarily for degradation of oxidized mitochondrial proteins that come into contact with free radicals in the respiratory chain (Whitaker et al., 2016), and interestingly has been positively selected for in Tibetan sheep (Wei et al., 2016). *Nfe2l1* (also known as *Nrf1*) is a transcription factor known to control a variety of processes including metabolic and proteasome homeostasis, and oxidative stress response, particularly through the glutathione synthesis pathway (Kim et al., 2016). Finally, the upregulated and highly respliced *Chchd4* encodes a redox-sensitive mitochondrial protein and a known positive regulator of hypoxia-inducible factor 1-alpha (Hif-1α). The hypoxic microenvironment found in tumors typically induces high expression of this hypoxia-responsive machinery, which then proceeds to promote the survivability and invasiveness of the tumor (Yang et al., 2012).

### Circuit Function and Gene Expression

Our study coupled two powerful techniques for probing the seasonal changes that occur in a very specific area of the brain: microelectrode-array electrophysiology and RNA sequencing. We observed altered behavior in nearly every electrophysiological metric measured, including action-potential kinetics that show an increase in depolarization charge transfer.

Our gene expression screen uncovered the upregulation of several candidate genes that could contribute to increased synaptic connectivity, outgrowth, and release, such as *Snap25, Nsf, Casr, Cplx2*, and *Map1b*. We also found several genes encoding ion-channel and pump subunits, most of which are heavily respliced, such as *Hcn3, Scn1b, Scn1a*, and *Atp1a3.* Either of these categories of genes could explain the winter-season increase in electrophysiological performance in general, and some results regarding action potential amplitudes and waveforms in particular. It makes sense that, in preparation for an environment where low temperatures would impede synaptic kinetics, key components of signal transmission systems would be fine-tuned to ensure reliable generation of breathing rhythms in such a vulnerable state. Future experiments will need to be performed to establish a definitive mechanistic link between gene regulation/splicing and circuit-level alteration of electrophysiological performance.

## Ethics Statement

All procedures were carried out in accordance with, and approved by the Basel-City Cantonal Veterinary Authority (Basel, Switzerland).

## Author Contributions

TR performed primary experimental, analysis and wrote the manuscript. JZ rendered the experimental assistance. FF and MO contributed analysis consulting. SB performed histological consultation. AH investigated the experiment.

## Conflict of Interest Statement

The authors declare that the research was conducted in the absence of any commercial or financial relationships that could be construed as a potential conflict of interest.

## Footnotes

1 www.mousemine.org

## Supplementary Material

FIGURE S1 Categories of genes ranked by select parameters. **(A)** Top 50 genes ranked by gene score. Green indicates an increase in the parameter indicated (column) for a particular gene (row). Red indicates a decrease. **(B)** Bottom 50 genes ranked by gene score. **(C)** Top 50 genes ranked by a DE factor produced by the SeqGSEA R package. **(D)** Top 50 genes ranked by a DS factor produced by the SeqGSEA R package. **(E)** Top 50 genes ranked by log-fold-change. **(F)** Bottom 50 genes ranked by log-fold-change. Most intense red indicates genes that are completely turned off. **(G)** Top 50 genes ranked by log-count-change. **(H)** Bottom 50 genes ranked by log-count-change. We then used the junctionSeq R package to determine DS from a select group of genes from our screen that were indicated to have a high level of DS. The package’s MA plot produced 1208 genes with a significant differential fold change (Figure 7A), a slightly higher but similar number of genes produced by the SeqGSEA screen. We selected three genes from Figure 6D (ion handling) and another three from Figure 6E (synapse structure) for further investigation into which exons are being differentially expressed. We found that Na+/K+ ATPase 1 α3 subunit (ATP1A3, Figure 7B) has three middle exons targeted for increased expression in the winter condition. K+ voltage-gated subfamily C (KCNC3, Figure 7C) exhibits what seems to be the swapping of a short for a long exon, whereas Na+ voltage-gated channel 1β (SCN1B, Figure 7D) eliminates its C-terminal exon during winter preparation. In the synaptic structure category of genes, both Synuclein-α (SNCB) and γ (SNCG) undergo elimination of C-terminal exons in winter (Figure 7E,F). Rabphilin-3A (RAB3A, Figure 7G) also eliminates a C-terminal exon, but swaps in two N-terminal exons in replacement.

FIGURE S2 Differential expression versus splicing for oxidative stress and metabolism genes. Count change versus DS for oxidative stress and metabolism genes. Select genes encoding for oxidative stress and metabolism genes are highlighted in red. Grey points indicate all 950 genes from the screen. Blue dashed lines represent neutral count change (vertical; zero log-fold-change) and the minimum DS score for significance (horizontal).

TABLE S1 Significant GO terms generated by RNA Seq. The left-most column lists standardized GO term generated by the 950 genes from Figure 6C. The *p*-value is the probability of a false positive as determined by the permutation screen described in Figure 6C. The ontology name is a brief description of the process. The right-most section contains the genes associated with producing the GO term in the same row. We further segregated the 950 genes in our screen by ranking independently by DE, DS, logarithm of fold change, and logarithm of count change. We reasoned that large count changes would represent genes that were highly expressed (such as structural components) and made a large contribution to the cell’s molecular makeup. Supplementary Figure S1 shows the highest ranked DE and DS genes, followed by the highest and lowest log-fold-change and log-count-change (Supplementary Figures 1C–H). Highest and lowest gene scores are shown for comparison (Supplementary Figures 1A–B).
