# Supplementary material for: Medullary Respiratory Circuit Is Reorganized by a Seasonally-Induced Program in Preparation for Hibernation
Source: Front Neurosci. 2019 Apr 26;13:376. doi: 10.3389/fnins.2019.00376 (PMC6497738; doi:10.3389/fnins.2019.00376)
Supplement: FIGURE S1 — Categories of genes ranked by select parameters. (A) Top 50 genes ranked by gene score. Green indicates an increase in the parameter indicated (column) for a particular gene (row). Red indicates a decrease. (B) Bottom 50 genes ranked by gene score. (C) Top 50 genes ranked by a DE factor produced by the SeqGSEA R package. (D) Top 50 genes ranked by a DS factor produced by the SeqGSEA R package. (E) Top 50 genes ranked by log-fold-change. (F) Bottom 50 genes ranked by log-fold-change. Most intense red indicates genes that are completely turned off. (G) Top 50 genes ranked by log-count-change. (H) Bottom 50 genes ranked by log-count-change. We then used the junctionSeq R package to determine DS from a select group of genes from our screen that were indicated to have a high level of DS. The package’s MA plot produced 1208 genes with a significant differential fold change (Figure 7A), a slightly higher but similar number of genes produced by the SeqGSEA screen. We selected three genes from Figure 6D (ion handling) and another three from Figure 6E (synapse structure) for further investigation into which exons are being differentially expressed. We found that Na+/K+ ATPase 1 α3 subunit (ATP1A3, Figure 7B) has three middle exons targeted for increased expression in the winter condition. K+ voltage-gated subfamily C (KCNC3, Figure 7C) exhibits what seems to be the swapping of a short for a long exon, whereas Na+ voltage-gated channel 1β (SCN1B, Figure 7D) eliminates its C-terminal exon during winter preparation. In the synaptic structure category of genes, both Synuclein-α (SNCB) and γ (SNCG) undergo elimination of C-terminal exons in winter (Figure 7E,F). Rabphilin-3A (RAB3A, Figure 7G) also eliminates a C-terminal exon, but swaps in two N-terminal exons in replacement. [file Data_Sheet_1.pdf]

Supplementary Figure 1

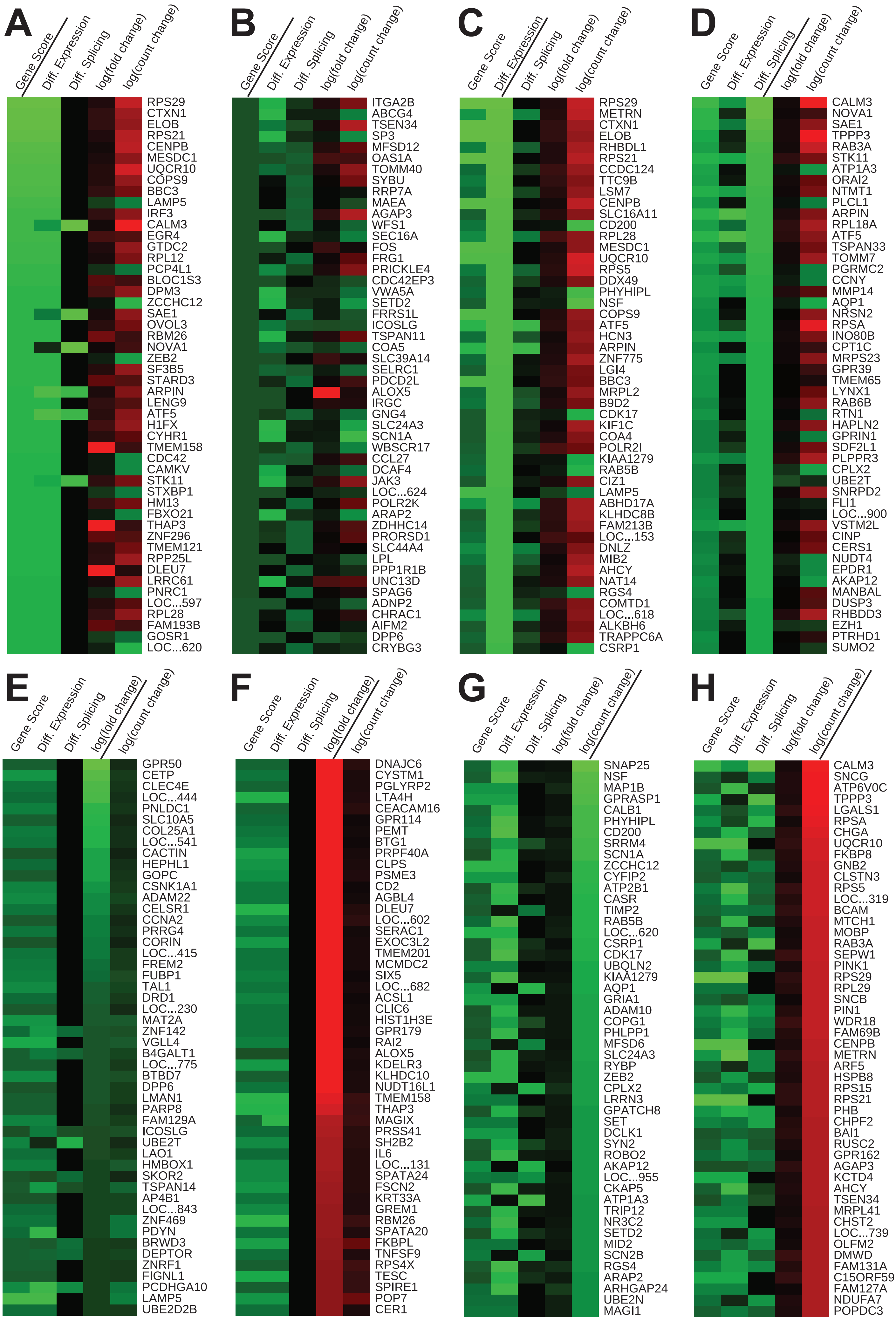

Supplementary Figure 2

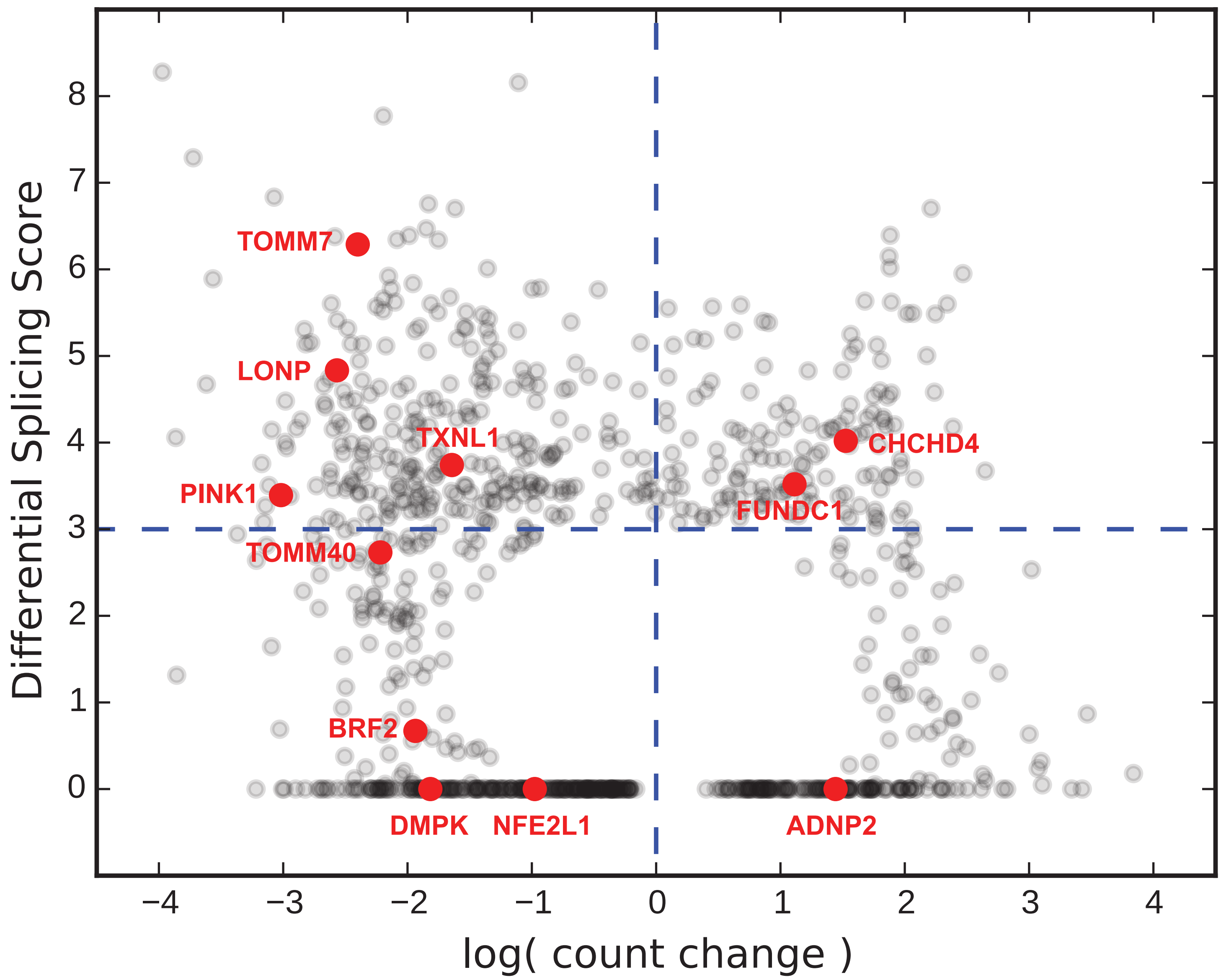

Supplementary Table 1

GO Term	p-value	Ontology Name	Genes
**Level 2**			
GO:0009986	0.014	cell surface	ADAM10 AMBP ARSB B4GALT1 BCAM CASR CD2 CLSTN1 CLSTN3 CORIN CX3CL1 DPP6 FLT3L GP5 GREM1 GRIA1 GRM7 HBEGF ITGA2B KCNH2 LGALS1 LPL LRFN3 LRRC8A NTRK2 P2RY12 PHB ROBO1 ROBO2 SCARB1 SLITRK6 TIMP2 TMEM8B TNFRSF1A TSPAN14 TSPAN33 TSPAN8
GO:0003735	0.000	structural constituent of ribosome	MRPL19 MRPL2 MRPL36 MRPL41 MRPS12 MRPS23 MRPS7 NDUFA7 RPL12 RPL15 RPL18A RPL28 RPL29 RPL30 RPL34 RPL35 RPL37 RPL38 RPS15 RPS21 RPS29 RPS4X RPS5 RPSA
GO:0016049	0.018	cell growth	ATP6V0E2 FUBP1 IL6 LAMTOR1 LAMTOR2 NDNF PRMT6
GO:0005622	0.037	intracellular	ABI3 ARF5 ARF6 ARL10 BACH2 CALB1 CAMKV CDC42 COPG1 CSTB DAB1 DCLK1 DEPTOR EVI5L FAM53A HSPB8 JAK3 KIF1C LGALS1 MAP1LC3B MID2 MRPL19 MRPL2 MRPL36 MRPS12 NMRK2 NOS2 NR3C1 PARP8 PITPNB RAB5B RAB6B RDH12 RHOU RILPL2 RNF114 RPL15 RPL28 RPL30 RPL38 RPS21 RPS29 RPS4X RPSA TRIM41 TRIM50 TWF2 UNC119B
GO:0030246	0.023	carbohydrate binding	BCAN CLEC2L CLEC4E CRYBG3 DGCR2 GALNT16 LGALS1 LMAN1 NPTX2 PFKM SIGLEC1 WBSCR17
**Level 3**			
GO:0001965	0.033	G-protein alpha-subunit binding	DRD1 GRIA1 PPP5C RGS4 RGS8
GO:0042802	0.019	identical protein binding	ABI3 AHCY APIP APLP2 BAG2 BAIAP2 C1QL4 CASP8 CCDC155 CEACAM16 CLDN3 ERBB3 FKBP8 FRS3 GALE GP1BB GPR50 GRIA1 HMBOX1 HSPB8 IIGP1 IRF3 ITGA2B ITPA JUN KCNH2 LMAN1 MAP1S MAT2A MID2 MSANTD3 NDEL1 PFKM PPARG PPP5C PSME3 PYCRL RABAC1 RASSF1 RBMX RILPL2 ROBO1 ROBO2 SDSL SH2B2 SNX1 SNX33 STXBP1 SYT3 TRIM41 TRIM50 UBE2G2 VWA1 WAS
GO:0048306	0.027	calcium-dependent protein binding	CALM3 CPLX2 DDX5 GRM7 NSMF SNAP25 SYN2 WFS1
GO:0043679	0.008	axon terminus	AQP1 CASR CHRM3 DRD1 GRM7 KCNC3 NTRK2 PDYN SNCB SNCG
GO:0032809	0.013	neuronal cell body membrane	AQP1 ATP2B1 KCNB1 KCNC3 RGS8 UNC5A
GO:0003707	0.007	steroid hormone receptor activity	NR0B1 NR1H3 NR3C1 NR3C2 PAQR7 PAQR9 PPARG RARA
GO:0030742	0.007	GTP-dependent protein binding	CDC42 GCH1 RAB3A RAB5B RAPGEF6
GO:0005496	0.014	steroid binding	NR3C1 NR3C2 PAQR7 PAQR9 PGRMC2
GO:0042493	0.046	response to drug	AQP1 ATP1A3 BLOC1S3 DGCR2 DRD1 FOS JUN KCNJ11 MAT2A PEMT SRP14
GO:0043195	0.043	terminal bouton	CALB1 CALB2 CPLX2 GRM7 NTRK2 PVALB RAB3A SNAP25 SNCB STXBP1 SYN2
GO:0043025	0.029	neuronal cell body	ATP1A3 BAIAP2 CALB1 CASK CASR CIB1 CPLX2 DAB1 DPP6 DRD1 EFHC1 GRIA1 GRIN3B GRM7 GRM8 HCN3 INHA KCNB1 KCNJ11 KCNN4 MAP1B MAP1LC3B MAP1S NRSN2 NTRK2 PDYN PLXDC1 PPP1R1B PPP5C PTK2B PVALB RARA ROBO1 RPSA SCN1A SNAP25 SNCB SNCG TIMP2 VGF
GO:0016021	0.013	integral component of membrane	ABCG4 ACSL1 ADAM10 ADAM22 ADCK2 AGPAT1 AGPAT2 AGPAT4 AIFM2 AKAP1 ALG10B APLP2 AQP1 ARV1 ASPHD2 ATP1A3 ATP2B1 ATP6V0C ATP6V0E2 B3GALT4 B3GAT1 B4GALT1 BCAM BEAN1 CABP7 CASR CCDC155 CD2 CD200 CDH9 CELSR1 CHRM3 CHST1 CHST2 CISD1 CLDN19 CLDN3 CLEC2L CLEC4E CLIC6 CLPTM1L CLSTN1 CLSTN3 COL25A1 COMTD1 CORIN CPT1C CTXN1 CX3CL1 CYSTM1 DGCR2 DIO1 DMPK DNAJC22 DPM3 DPP6 DRD1 ECEL1 EMC10 ERBB3 FAM134B FAM155A FAM171A2 FAM210B FAM69B FIBCD1 FKBP8 FLT3L FREM2 FRRS1L FUNDC1 FYCO1 GALNT16 GJB1 GOSR1 GP1BB GPBAR1 GPR108 GPR162 GPR39 GPR50 GPR88 GRIA1 GRIN3B GRM7 GRM8 GXYLT1 HBEGF HCN3 HEPHL1 HS3ST3A1 IER3IP1 ITGA2B KCNB1 KCNC3 KCNH2 KCNJ11 KCNJ9 KCNN4 KDELR3 LAMP5 LHFPL3 LMAN1 LRFN3 LRRC4 LRRN3 MANBAL MCOLN3 METTL23 MFSD12 MFSD6 MMP14 MOSPD3 MTCH1 MXRA7 NAT14 NAT8L NCAM2 NFE2L1 NIPAL3 NRSN2 NTRK2 ORAI2 P2RY12 P2RY6 PAQR4 PAQR7 PAQR9 PEMT PEX2 PGRMC2 PINK1 PLPP5 PLPPR3 PLXDC1 PLXNA3 PNLDC1 POPDC3 PRRG4 PTAFR PTCHD1 QRFPR RABAC1 RHBDD3 RHBDL1 RNF182 ROBO1 ROBO2 RTN1 S1PR1 SCARB1 SCN1A SCN1B SCN2B SCNN1A SERAC1 SERP1 SERP2 SIGLEC1 SIRPA SLC10A5 SLC10A7 SLC16A11 SLC16A8 SLC1A5 SLC24A3 SLC39A1 SLC39A14 SLC44A4 SLITRK3 SLITRK6 STARD3 STEAP4 STXBP5L SURF4 SUSD2 SYBU SYT3 TM7SF3 TMCC1 TMEM114 TMEM121 TMEM141 TMEM158 TMEM168 TMEM184B TMEM201 TMEM65 TMEM88B TMEM8B TMEM9 TNFRSF1A TNFSF9 TOMM40 TOMM7 TOR1AIP1 TSPAN11 TSPAN14 TSPAN33 TSPAN8 TTYH2 UBIAD1 UCP1 UCP2 UNC5A VSTM4 WBSCR17 WFS1 ZDHHC14 ZDHHC9
GO:0000149	0.043	SNARE binding	CPLX2 EXOC3L2 GOSR1 KCNB1 SNAP25 STXBP1 SYT3
**Level 4**			
GO:0016363	0.046	nuclear matrix	ALOX5 ANP32A ATN1 CASK MAEA NSMF PRKCZ PRPF40A
GO:0006914	0.040	autophagy	ARSB ATG4A FAM134B FUNDC1 GOPC LRSAM1 MAP1LC3B NRBF2 PINK1 RAB12 RAB33B STK11 UBQLN2
GO:0005840	0.001	ribosome	MRPL19 MRPL2 MRPL36 MRPL41 MRPS12 MRPS23 MRPS7 RPL12 RPL15 RPL18A RPL28 RPL29 RPL30 RPL38 RPS15 RPS21 RPS29 RPS4X RPS5 RPSA TMA16
GO:0046928	0.001	regulation of neurotransmitter secretion	CELSR1 GRM8 KCNC3 NGF NTRK2 SNCG
GO:0007030	0.042	Golgi organization	CDC42 CSNK1A1 GORASP1 KIFC3 LMAN1 PLEKHM2 RAB30 SURF4
GO:0034599	0.037	cellular response to oxidative stress	ADNP2 BRF2 CHCHD4 LONP1 NFE2L1 PINK1 TXNL1
GO:0070412	0.003	R-SMAD binding	DDX5 FOS FOXH1 JUN RANBP3 ZEB2
GO:0043401	0.022	steroid hormone mediated signaling pathway	BMP7 NR0B1 NR1H3 NR3C1 NR3C2 PPARG RARA
GO:0032590	0.024	dendrite membrane	ATP2B1 GRIA1 KCNB1 KCNC3 LAMP5
GO:0042127	0.01	regulation of cell proliferation	ATF5 B4GALT1 BCL6B CDK6 CIB1 ERBB3 IL6 INHA MAFG MATK NOS2 NR3C1 NR3C2 PIN1 PTK2B RNF126 TAL1 TNFRSF1A TNFSF9 TOPORS
GO:0030424	0.026	axon	ADAM22 AQP1 ATP1A3 CALB1 CASR CIB1 CPT1C DRD1 GRM7 HCN3 KCNB1 MAP1B MAP1LC3B MAP3K12 NCAM2 NDEL1 NRN1L NTRK2 PINK1 PTK2B PVALB RAB3A ROBO1 SCN1A SNAP25 SNCG TNFRSF1A
GO:0005634	0.047	nucleus	ADAM10 ADNP2 AGAP3 AHCY ALKBH6 ALOX5 ANKRD23 ANKRD54 ANP32A APLP2 AQP1 ARL6IP4 ATF5 ATN1 ATP1A3 ATP2B1 AUTS2 B9D2 BACH2 BCL6B BRF2 BRWD3 BTBD7 BTG1 CACTIN CALB1 CALB2 CALM3 CASK CASP3 CASP8 CASR CBR1 CBX4 CBX5 CCDC106 CCDC155 CCNA2 CCNK CCNY CDC34 CDK17 CDK6 CDKL1 CENPB CHRAC1 CIB1 CINP CIZ1 CLDN19 CLIC6 CLSTN1 COMMD10 COPS9 CSNK1A1 CSRP1 CSTB CSTF2 CYFIP2 DCLRE1B DDX5 DEDD2 DGKZ DGUOK DMPK DNAJB1 DRD1 DUSP3 DYRK3 EAF1 EGR4 ELL3 ELOB EPS15L1 ERBB3 EXOSC4 EZH1 FAM193B FAM53A FAM83G FIGNL1 FIGNL2 FLI1 FOS FOXH1 FOXJ1 FRA10AC1 FRG1 FUBP1 GCH1 GLIS1 GNA13 GNL1 GPR88 GPRASP1 H1FX H2AFX HINT3 HIST1H3E HMBOX1 HMG20B HOXD1 HR HSF4 HSF5 HSPB2 HSPB8 IIGP1 INO80B IQSEC1 IRF2BPL IRF3 JUN KLHDC10 LDOC1 LGALS1 LONP1 LSM4 LTA4H MAEA MAFG MAGI1 MAGI3 MAP1S MBD5 MED1 MED25 MED27 MED29 MED31 MEF2A METTL22 MEX3B MGRN1 MIER2 MMP14 MPG MRPL19 MVB12A MYCL MZF1 NACC1 NFE2L1 NHLH2 NIP7 NME2 NOS2 NOVA1 NR0B1 NR1H3 NR3C1 NR3C2 NRBF2 NSMF NTMT1 NUDT16L1 NUDT4 OAS1A OLFM2 OVOL3 PAF1 PATZ1 PCIF1 PDCD7 PDLIM7 PHB PHLPP1 PIN1 PINK1 PLXNA3 PNMA1 PNRC1 POLR2E POLR2I POLR2K POP7 PPARG PPIL2 PPP1R1B PPP5C PQBP1 PRKCZ PRMT6 PRPF40A PRPF6 PSMD7 PSMD9 PSME3 PTK2B PVALB RAD17 RAD51D RANBP3 RARA RASSF1 RBM42 RBMX RDM1 RFWD2 RFXANK RGS4 RGS8 RNF114 RNF126 RNPS1 RPL12 RPL15 RPL30 RPP25L RPRD1B RPS4X RPSA RUSC1 RYBP SAE1 SAFB SCAF11 SCAND1 SDR39U1 SERTAD1 SET SETD2 SF3A1 SF3B4 SF3B5 SIN3B SIVA1 SIX5 SKOR2 SMU1 SNRNP35 SNRPD2 SOX12 SP1 SP3 SPATA24 SRP14 SRRM4 SS18L1 ST18 STK11 SUMO2 SURF6 SWSAP1 SYMPK TAL1 TCF15 TCF20 TESC TIGD5 TMA16 TMEM201 TMEM8B TNFRSF1A TOPORS TOR1AIP1 TOX2 TRIB1 TRIM41 TRIP12 TSEN34 TSHZ2 TSSK6 TUBB2A TXNDC9 TXNL1 U2AF1L4 U2AF2 UBE2N UBE2T UBIAD1 UBOX5 UBQLN2 UBXN6 USF2 VGLL4 WBSCR22 WDR18 XPC YPEL4 ZBTB22 ZBTB43 ZCCHC12 ZEB2 ZMAT5 ZNHIT1 ZSCAN21
**Level 5**			
GO:0022627	0.014	cytosolic small ribosomal subunit	RPS15 RPS21 RPS29 RPS4X RPS5 RPSA
GO:0017075	0.012	syntaxin-1 binding	CPLX2 NSF SNAP25 STXBP1 SYBU
GO:0022625	0.000	cytosolic large ribosomal subunit	RPL12 RPL15 RPL18A RPL28 RPL29 RPL30 RPL34 RPL35 RPL37 RPL38 SURF6
GO:0048487	0.024	beta-tubulin binding	AKAP1 B4GALT1 MAP1S NDEL1 SNCB SNCG
GO:0019228	0.042	neuronal action potential	DPP6 DRD1 GPR88 GRIA1 SCN1A
GO:0007616	0.049	long-term memory	CALB1 GRIA1 NTRK2 PRKCZ SNAP25
GO:0005689	0.000	U12-type spliceosomal complex	LSM7 PDCD7 RBM41 SF3B4 SF3B5 SNRNP35 SNRPD2 ZMAT5
GO:0030307	0.026	positive regulation of cell growth	ADAM10 ADNP2 CIB1 EXOSC4 HBEGF MMP14 NGF PTK2B SLC44A4
GO:0071300	0.021	cellular response to retinoic acid	ADNP2 AQP1 PTK2B RARA TESC TWF2
GO:0006412	0.001	translation	EIF4E3 FARS2 MRPL19 MRPL2 MRPL36 MRPL41 MRPS12 MRPS23 MRPS7 RPL12 RPL15 RPL18A RPL28 RPL29 RPL30 RPL34 RPL35 RPL37 RPL38 RPS15 RPS21 RPS29 RPS4X RPS5 RPSA SARS2
GO:0031307	0.011	integral component of mitochondrial outer mem.	DMPK FUNDC1 PINK1 TOMM40 TOMM7
GO:0061630	0.013	ubiquitin protein ligase activity	CDC34 FBXO30 KCMF1 LRSAM1 MED1 MED27 MED31 MGRN1 MIB2 PEX2 PPIL2 RFWD2 RNF114 RNF126 TOPORS TRIP12 UBE2G2 UBE2N UBE2T UBOX5 ZNRF1
**Level 6**			
GO:0042593	0.032	glucose homeostasis	GPR39 IL6 KCNB1 MBD5 PFKM PPARG SH2B2 STK11 STXBP5L VGF WFS1
GO:0071347	0.034	cellular response to interleukin-1	CACTIN CX3CL1 HYAL3 IL6 RBMX
GO:0000028	0.002	ribosomal small subunit assembly	MRPS7 RPS15 RPS5 RPSA RRP7A
GO:0030218	0.015	erythrocyte differentiation	CASP3 DYRK3 JAK3 NFE2L1 SP3 TAL1
GO:0007601	0.039	visual perception	CRYBA4 GPR179 HSF4 NGB OLFM2 PDE6D RDH12 SLITRK6 WFS1
GO:0008380	0.020	RNA splicing	ARL6IP4 CACTIN DCAF4 DDX5 FRG1 LSM4 PQBP1 PRPF40A PRPF6 RBMX RNPS1 SCAF11 SF3A1 SF3B4 SF3B5 SNRNP35 SNRPD2 SRRM4 U2AF1L4 U2AF2 ZMAT5
**Level 7**			
GO:0051291	0.024	protein heterooligomerization	CLDN3 GCH1 HR KCNH2 MAT2A PPP5C PRKCZ SEPT9 STK11 TNFRSF1A
GO:0048814	0.008	regulation of dendrite morphogenesis	NSMF PQBP1 ROBO1 SKOR2 STK11
GO:0005272	0.024	sodium channel activity	HCN3 SCN1A SCN1B SCN2B SCNN1A
GO:0043524	0.042	negative regulation of neuron apoptotic process	ERBB3 FAM134B JUN NDNF NGF NTRK2 PIN1 PINK1 PTK2B SET SNCB STXBP1 VSTM2L WFS1
GO:0043525	0.043	positive regulation of neuron apoptotic process	BBC3 CASP3 CDC34 CDC42 JUN NR3C1 PIN1 TNFRSF1A
GO:0050772	0.009	positive regulation of axonogenesis	METRN NTRK2 ROBO1 ROBO2 STK11 ZEB2
GO:0003924	0.034	GTPase activity	AGAP3 ARF6 CDC42 GCH1 GNA11 GNA13 GNB2 GNG4 GNL1 GPN3 IIGP1 MMAA RAB12 RAB30 RAB33B RAB3A RAB5B RAB6B RHOU TUBB2A
GO:0000398	0.033	mRNA splicing, via spliceosome	LSM4 NOVA1 PRPF40A PRPF6 RBM41 SF3A1 SF3B5 SNRNP35 U2AF1L4 U2AF2
GO:0032024	0.038	positive regulation of insulin secretion	CASK CASR GPR39 PFKM PSMD9 SERP1
